# Sustainable and innovative biological control strategies against *Pseudomonas syringae* pv*. tomato, Pseudomonas savastanoi* pv*. phaseolicola* and *Xanthomonas* spp. affecting vegetable crops: a review

**DOI:** 10.3389/fpls.2025.1536152

**Published:** 2025-04-30

**Authors:** Davide Giovanardi, Enrico Biondi, Nina Biondo, Nicolás Quiroga, Francesco Modica, Gerardo Puopolo, Set Pérez Fuentealba

**Affiliations:** ^1^ Department of Life Sciences, University of Modena and Reggio Emilia, Reggio Emilia, Italy; ^2^ Department of Agricultural and Food Sciences (DISTAL), Alma Mater Studiorum - University of Bologna, Bologna, Italy; ^3^ Institute of Agri−Food, Animal and Environmental Sciences (ICA3), Universidad de O’Higgins, San Fernando, Chile; ^4^ Center Agriculture Food Environment (C3A), University of Trento, San Michele all’Adige, Italy

**Keywords:** bacterial plant diseases, vegetables, essential oils, microbiological control agents, bacteriophages

## Abstract

Genera *Pseudomonas* and *Xanthomonas* include bacterial species that are etiological agents of several diseases of major vegetable crops, such as tomato, pepper, bean, cabbage and cauliflower. The bacterial pathogens of those genera may cause severe crop damage, leading to symptoms like leaf spots, wilting, blights, and rotting. These plant pathogens can affect propagation materials and spread rapidly through plant tissues, contaminated soils, or water sources, making them challenging to control using conventional chemical products alone. Biopesticides, such as essential oils (EOs), are nowadays studied, tested and formulated by employing nano- and micro-technologies as innovative biological control strategies to obtain more sustainable products using less heavy metal ions. Moreover, there is a growing interest in exploring new biological control agents (BCAs), such as antagonistic bacterial and fungal species or bacteriophages and understanding their ecology and biological mechanisms to control bacterial phytopathogens. These include direct competition for nutrients, production of antimicrobial compounds, quorum quenching and indirect induction of systemic resistance. Optimisation of the biocontrol potential goes through the development of nanoparticle-based formulations and new methods for field application, from foliar sprays to seed coatings and root inoculation, aimed to improve microbial stability, shelf life, controlled release and field performance. Overall, the use of biological control in horticultural crops is an area of research that continues to advance and shows promising potential. This review aims to provide an in-depth exploration of commercially accessible biocontrol solutions and innovative biocontrol strategies, with a specific focus on the management of bacterial diseases in vegetable crops caused by *Pseudomonas* and *Xanthomonas* species. In this article, we highlighted the advancements in the development and use of EOs and other BCAs, emphasizing their potential or shortcomings for sustainable disease management. Indeed, despite the reduced dependence on synthetic pesticides and enhanced crop productivity, variable regulatory frameworks, compatibility among different BCAs, and consistent performance under field conditions are among the current challenges to their commercialization and use. The review seeks to contribute valuable insights into the evolving landscape of biocontrol in vegetable crops and to provide guidance for more effective and eco-friendly solutions against plant bacterial diseases.

## Graphical Abstract

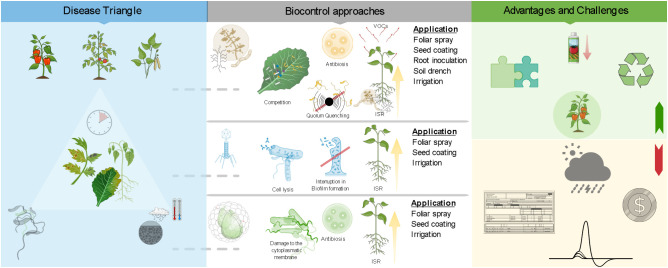

## 1 Introduction

The world’s population will grow by 39% over the next two decades, reaching 9.1 billion by 2050 (www.fao.org). Thus, it will be necessary to increase world food production by 60% as there will be more people to feed, and agriculture has to become more productive. Vegetables are essential sources of the micronutrients needed for healthier diets and enable consumers to tap the nutritional power of vegetables (Schreinemachers et al., 2018). Many economically important vegetable crops, including tomato (*Solanum lycopersici* L.), pepper (*Capsicum annuum* L.), beans (*Phaseolus vulgari*s L.), cabbage (*Brassica oleracea* var. *capitata* L.) and cauliflower (*Brassica oleracea* var. *botrytis* L.) suffer from bacterial infections, which are thought to account for yield losses of 5–10% (Holtappels et al., 2021). Gram-negative bacterial species belonging to *Pseudomonas* and *Xanthomonas* genera represent the most relevant and destructive plant pathogenic bacteria (Mansfield et al., 2012).

Bactericides, copper-based products, and/or antibiotics (*e.g.*, streptomycin, kasugamycin, tetracyclines) have a long history and are still the main strategies readily available and used for bacterial disease management. However, chemical control of bacterial pathogens still results in a problematic and even ineffective control due to their frequent polycyclic nature and the lack of systemic antibacterial substances (Oerke, 2006), therefore not providing a solution to plant infection or disease eradication (Dewdney and Graham, 2017). The use of chemical pesticides is also associated with a loss in their efficacy because of the co-selection of antimicrobial resistance (AMR) to specific active substances, such as copper compounds and antibiotics (Miller et al., 2022), reported mainly as prevalent in *Pseudomonas* spp. and *Xanthomonas* spp (Cooksey, 1994; Islam et al., 2024). Moreover, chemical pesticide use can result in potentially detrimental consequences, impacting the human population, environmental health, and ecology of the phytobiome. Thus, there is a need for alternative solutions that are durable, sustainable, accessible to farmers, specific in their target, and environmentally friendly, mainly where conventional approaches are limited or compromised.

One possible alternative is represented by biopesticides, defined as “pesticides derived from natural materials that can manage pests by specific biological effects or actions” (Koul, 2023). This definition allows to include under the “biopesticide umbrella” the three major classes listed by the US Environmental Protection Agency (EPA): 1) biochemicals (*i.e.*, pheromones and botanicals), 2) microbes (*i.e.*, bacteria, fungi, viruses, and protozoans), and 3) plant-incorporated-protectants (PIPs, *i.e.*, genetically engineered crops) (https://www.epa.gov/ingredients-used-pesticide-products/what-are-biopesticides#advantages).

Botanicals (*e.g.*, plant extracts, EOs) have a broad antibacterial spectrum that inhibits the growth of phytopathogens through the interaction of their hydrophobic components with the lipids present in the cell membrane of microorganisms, resulting in metabolic damages and bacterial cell death (Da Silva et al., 2021).

The hydrophobic nature of EOs is conferred by several molecules such as terpenes and terpenoids, but also other compounds as alcohols, aldehydes, aliphatic hydrocarbons, acyclic esters or lactones; this nature allows the interaction against the cytoplasmic membrane by inducing alterations of it, by increasing cell permeability, that leads to leakage of the cell contents, or by causing alterations in cell structure and functionality (Nazzaro et al., 2013).

In particular, EOs are a mixture of volatile oils produced as a secondary metabolite in different aromatic plant species (Bakkali et al., 2008), showing the potential as safe, biodegradable alternatives to conventional toxic chemical bactericides in agriculture (Assadpour et al., 2024). Recent studies have proven the antimicrobial effectiveness of EOs against antibiotic-resistant bacteria (ARB), candidating them as a potential sustainable solution to the management of ARB phytopathogens (Di Vito et al., 2019). Moreover, EOs may play important roles in the plant immune system, acting either as antimicrobials or as signal molecules for the activation of the plant defenses (Choudhary et al., 2021).

Besides, the effectiveness of microbiological control agents (*m*BCAs) in controlling plant diseases is characterized by multiple modes of action: (*a*) competition for resources (*e.g.*, oxygen, carbon, nitrogen, and other essential resources); (*b*) antibiosis via effects of toxic secondary metabolites; (*c*) hyperparasitism, where the antagonist acts as a predator and exploits the pathogen as a prey; (*d*) induced systemic resistance (ISR) *in planta* against invading plant pathogens; (*e*) stimulation by better nutrient absorption and/or by affecting plant hormone pathways (*e.g.*, rhizosphere bacteria and fungi).

Although bacteriophages (*phage*BCAs) are viruses that specifically infect and replicate in bacteria as antimicrobial agents, they should not be considered conventional *m*BCAs together with other true microorganisms (*i.e.*, bacteria and fungi) (Stefani et al., 2021). *phage*BCAs can replicate and spread through lytic life cycles, leading to the degradation of bacterial hosts and exhibiting a high degree of specificity and persistence/proliferation in the environment (Buttimer et al., 2017; Rabiey et al., 2020). These are the most important determinants that confer control characteristics to *phage*BCAs as a promising multifaceted tool for the management of bacterial disease (Farooq et al., 2022).

It is widely accepted that the use of these biopesticides does not guarantee the level of crop protection achieved using a single formulated antibiotic compound. However, growers could employ several compounds of different origins or modes of action to achieve such a control level by using only biocontrol strategies during the spread of the epidemic. Moreover, it is worth mentioning that the real long-term advantage of the use of more sustainable bioproducts is their multifaceted nature. Indeed, their application could significantly reduce the possibility of upcoming bacterial resistance due to their several modes of action.

However, there are other various limitations to the use of biopesticides, compared to the use of synthetic pesticides in agriculture, such as: lower efficacy, that is highly dependent on environmental field conditions (*e.g.*, heat, UV light, desiccation), and a slower rate in the control of plant diseases, by limiting pathogen population through multiple modes of action but not a complete control (Ayilara et al., 2023). Besides, the complexity of the regulatory framework for registration, the decreased availability in the market, and the still wide skepticism of these products by farmers (Stefani et al., 2021).

## 2 Tomato and pepper*, Fabaceae* and *Brassicaceae* (cabbage and cauliflower): *Pseudomonas* and *Xanthomonas* bacterial diseases and their management

### 2.1 Tomato and pepper

Tomato and pepper are among the most cultivated vegetables crops worldwide, with over seven million hectares of cultivated area and more than 186 and 37 million metric tons of tomato and pepper produced, respectively (FAO - Food and Agriculture Organization of the United Nations, 2024). Among ​the most challenging bacterial diseases of pepper and tomato, bacterial spot and bacterial speck can cause significant reductions in field crop yields, especially if the infection appears early in the vegetative season (Ji et al., 2006); additionally, their causal agents are seed-borne and seed-transmitted, thus posing specific threat to the seed industry. Bacterial spot caused by *Xanthomonas vesicatoria* (Xv), *Xanthomonas euvesicatoria* pv. *euvesicatoria* (Xee), *Xanthomonas euvesicatoria* pv. *perforans* (Xep) and *Xanthomonas hortorum* pv. *gardneri* (Xhg) is a worldwide disease causing yield losses of up to 50% on tomatoes under warm and rainy weather (Buttimer et al., 2017). Xanthomonads enter tomato plants primarily through stomata, lenticels, and wounds, causing necrotic lesions on leaves with a polygonal shape. On fruits, symptoms are scab-like, raised, and whitish lesions, leading to decreased market value (EPPO, 2023). These causal agents are included in the A2 list of quarantine pathogens of the European and Mediterranean Plant Protection Organization (EPPO A1 and A2 Lists of Pests Recommended for Regulation as Quarantine Pests PM 1/2(32); https://gd.eppo.int/download/standard/2/pm1-002-32-en_A1A2_2023.pdf).

*Pseudomonas syringae* pv. *tomato* (Pst), the causal agent of bacterial speck, is categorized as a quarantine pest in Mexico, Egypt, Jordan, and China (https://gd.eppo.int/taxon/PSDMTM/categorization). Bacterial speck bacterial speck is more severe under cool (*i.e.*, between 18 and 25°C) and humid conditions, in which typical symptoms show small and dark leaf lesions, usually surrounded by a chlorotic halo that is provoked by the coronatine toxin and necrotic spots along stems and on fruits. Pst cells can evade the plant as exudates from necrotic lesions and spread around (Xhemali et al., 2024).

Pst, Xv, Xee, Xep and Xhg are widely distributed in different geographical regions, probably as the result of trading infected seeds or transplants. Thus, the management strategy should include using pathogen‐free, certified seed or disease‐free transplants (Xhemali et al., 2024). In addition, the removal of potential inoculum sources, such as volunteer plants and infected host plants, should be carried out promptly. Field isolation from infected host plants in close proximity, accompanied by sanitation, physical removal and disposal of diseased crop material, and crop rotation with non‐hosts, should be followed as Integrated Pest Management (IPM). However, the most common disease control strategies for both bacterial diseases are based on preventive application of copper-based products, alone or in combination with dithiocarbamate fungicides and antibiotics (where allowed), which poses a severe threat to human health, environment and the development of copper and/or antibiotic-resistant strains (Lamichhane et al., 2018; Šević et al., 2019). An additional control method of Pst race 0 is represented by the use of commercial tomato cultivars carrying the resistance gene *Pto*, which interacts with the avirulence gene of the plant pathogen (*e.g.*, *avrptoB* in Pst DC3000) by limiting its spread during an epidemic occurrence (Martin et al., 1993). Recently, a study concerning the characterization of the *hrpZ* gene, present in the hrc/hrp pathogenicity domain, was carried out by analyzing Pst strains isolated in different regions in Egypt performed pathogenicity investigations to study the level of virulence with specific RFLP-PCR, along with molecular marker system, and correlated to the development of the symptoms (El-Fatah et al., 2024).

On the contrary, commercial pepper and tomato varieties exhibiting complete resistance to bacterial spot are not available (Adhikari et al., 2020). In addition to qualitative resistance available against Pst race 0 strains mentioned above, quantitative resistance against Pst race 1 strains have been identified in wild tomato *Solanum habrochaites* accessions LA1777 and LA2109, and *Solanum lycopersicoides* accession LA295 (Mazo-Molina et al., 2020; Thapa et al., 2015). For bacterial spot, quantitative trait loci (QTL) associated with resistance were also identified in wild *Solanum pimpenellifolium* accession LA2533 or cultivated tomato relatives *Solanum lycopersicum* var. *cerasiformae* PI114490, tomato lines Hawaii 7998 and Hawaii 7981 (Hassan et al., 2024; Sharma and Bhattarai, 2019). More recently, the breeding between two tomato varieties, the susceptible line Ohio 88119 and the resistant line Ohio 9834 carrying the resistance locus (Rx3), was used to locate the locus responsible for the bacterial spot resistance against the race T1 in Xee (Meng et al., 2022). However, complete resistance has yet to be obtained because it is impeded by the emergence of Pst race 1 or by as many as four Xanthomonad species on tomato. Moreover, overcoming the identified resistant germplasm, multi-genic control of the resistance, linkage drag, non-additive components of the resistance, and the negative correlation between fruit quality and disease resistance have made the introgression of resistance even more challenging (Adhikari et al., 2020).

### 2.2 Fabaceae

The *Fabaceae* family is one of the most important vegetables that are consumed by people from every part of the world, among which beans are the third legume species with the highest economic relevance with an annual global yield of over 27 million metric tons (Fernandes Gomes et al., 2020; FAO - Food and Agriculture Organization of the United Nations, 2024).

Among the bacterial plant pathogens, *Pseudomonas savastanoi* pv. *phaseolicola* (Psph), *Xanthomonas axonopodis* pv. *phaseoli* (Xaph) and *Xanthomonas citri* pv. *fuscans* (Xcf) are the causal agents of halo blight, common blight and fuscous blight, respectively. These are the most important pathogens concerning direct crop damage and their further dissemination at long distances through bean seeds (Agarwal and Sinclair, 1997). Psph is a serious seed-borne pathogen that needs a low optimal temperature (less than 25°C) to survive and start the pathogenic process. Due to its rapid spread, even low levels of Psph primary infection can result in severe epidemics at optimal weather conditions (Schaad et al., 1995). The symptoms start from water-soaked lesions/spots on leaves, pods, and stems that develop in yellowish haloes on leaves (Arnold et al., 2011). Seeds are often asymptomatic, but they might carry primary inoculum sources (latent infections). Looking at Xanthomonads, Xaph and Xcf are seed-borne severe pathogens favored, in direct crop damage, by higher temperature (28-30°C). The symptomatology affects all the aerial parts of the bean plant, such as leaves, pods, and stems. These plant pathogenic bacteria provoke water-soaked spots that become necrotic, surrounded in the leaves by a yellow halo; even seeds may be symptomatic, showing lesions distributed all over the coat or close to the hilum area. The latent infections on seeds are the most dangerous because of the bacterial spread at long distances, as it happens for Psph (EFSA Panel on Plant Health (PLH), 2014).

In the field, in case of an epidemic spread, the control of these pathogens is mainly achieved through the application of copper-based products or antibiotics, where they are allowed. Still, the diagnostic analyses of bean seeds represent the most effective method to avoid infections that might start from contaminated/infected seeds (EFSA Panel on Plant Health (PLH), 2014) to have pathogen-free propagation material. In addition, as concerns the control of Psph, breeding for resistance could be another effective method to control that pathogen: based on gene-for-gene interactions, Psph is divided into nine races depending on the presence of different *avr* genes that interact with R genes (NLR resistance genes) of the host plant. Indeed, not all bean cultivars are susceptible to Psph (Arnold et al., 2011). The gene-for-gene interaction regarding Xaph is still being studied, and it involves the proposed dominant gene *xap-1* (Zapata et al., 2011). Thus, to date, there is no complete data on resistant bean cultivars.

### 2.3 *Brassicaceae* (cabbage and cauliflower)

The *Brassicaceae* family, which includes approximately 3.700 species, produces ornamental flowers, edible vegetables, and oilseed plants. It represents one of the ten most economically important vegetables in world agricultural and food markets, where cabbages and cauliflowers are among the most produced brassica with an annual global yield of *ca*. 73 and 26 million metric tons, respectively (FAO - Food and Agriculture Organization of the United Nations, 2024).

Black rot, caused by *Xanthomonas campestris* pv. *campestris* (Xcc) is considered one of the most important and destructive diseases affecting the quality and yield of *Brassicaceae*, in particular for the Brassica family, where it can reduce cabbage yield from 50 to 60% annually (Kong et al., 2021). Xcc is primarily a seed-borne disease, but this pathogenic bacterium can also be transmitted by wounding, water splash irrigation, or wind-driven rain. Here, a critical epidemiological aspect of *Xcc* is played by the possible rapid spread in transplants with the sprinkling irrigation systems used by plant growers. Moreover, Xcc can survive in the soil associated with plant debris for up to two years in the case of harsh stem residues and cold temperatures (Gazdik et al., 2021), persisting therefore between brassica crop rotations and acting as a source of primary *inoculum*. Under conducive environmental conditions (*i.e.*, high humidity and temperatures of 25–30°C), Xcc *inoculum* can enter the host plant vascular system through hydathodes or wounds along the leaf margin, causing V-shaped necrotic lesions (Köhl and van der Wolf, 2005). In most cases, Xcc moves systemically throughout the plant, causing leaf wilting, rot and, in case of severe infections, plant death (Greer et al., 2023). Several preventive agricultural practices and cultural methods (*e.g.*, certified and treated seeds; Brassica cultivation in 3-year field rotations; cleaning and sterilization of field equipment; crop residue management; elimination of other host plants of the pathogen), similar to those used for Xanthomonads and Pst, have been reported for controlling Xcc (Vicente and Holub, 2013).

On the contrary, the use of chemical pesticides in disease management has been shown to be often ineffective because they are applied when symptoms are visible and the disease is already established (Liu et al., 2022). Resistant cultivars represent one of the most effective approaches to control black rot, reducing the overall cost and chemical pollution. Nevertheless, the complex differentiation of Xcc in 11 physiological races, where races 1 and 4 are the most virulent and widespread, hampers the development of Xcc-resistant breeding lines. This is because only a few germplasms have clear race-specific resistance, and for cabbage, for example, the resources with high resistance are rare (Kong et al., 2021). Concerning the host range, in 2014, several Xcc strains were isolated from winter oilseed rape (*Brassica napus* L.); such strains from winter oilseed rape exhibited greater genetic diversity and had higher specificity towards this host isolates from the other brassicas (Jelušić et al., 2021).

## 3 Current status and legislation of biopesticides

During the last decades, evidence of significant advances in biopesticides and their applications is highlighted by the constant growth of the global biopesticides market. It reached a value of US$ 6.7 billion in 2023, which is expected to touch a value of US$ 13.9 billion by 2028, with a compound annual growth rate (CAGR) of 15.9% during 2023 - 2028 (https://www.marketsandmarkets.com/Market-Reports/biopesticides-267.html). However, it still represents about 10% of the global chemical pesticide market, estimated at around $79.3 billion in 2023.

Currently, there are hundreds of registered biopesticides worldwide, with more than 200 in the USA, 60 in the EU, 300 in China, and 400 in India (Chakraborty et al., 2023). Marrone (2024) reported that 396 out of 567 registered biopesticides in Brazil are used by conventional growers (among which 60.4% are microbials and 9% are botanicals). These data highlight that biopesticide development becomes an essential component of the integrated pest management (IPM) concept. However, their registration framework continues to be very challenging within the biopesticide industry, often using the same process as conventional chemical pesticides. Laws and policies regulating pesticide development, registration and use vary from country to country (*e.g.*, USA, UE, Cina, India, Brazil), with a non-uniform model that can simplify their registration process and promote the use of biopesticides (Arora et al., 2016). Different global agencies, such as the International Organization for Biological Control (IOBC), the European and Mediterranean Plant Protection Organization (EPPO) and the Organization for Economic and Co-operative Development (OECD), made efforts to provide some flexibility to biopesticide regulation. However, progress towards harmonization and work sharing still required progress through the development of guidance and working documents.

The USA has a simpler pesticide regulatory process than many other countries, such as Europe, with a distinct procedure for biopesticides and chemicals. In the United States, biopesticides are registered under the Pesticide Registration Improvement Act (PRIA) by involving the Environmental Protection Agency (EPA) and the Food and Drug Administration (FDA). The process has a lower cost and faster timeline than chemicals (~ 1–2 years to approve a new active biopesticide ingredient), a lower submission fee for small businesses, allowing continued innovation and more products on the market (Marrone, 2023). On the other hand, EU legislation for placing on the market biopesticides is not treated as a specific category (European Commission, 2009), but two categories of plant protection products (PPPs) are recognized: chemical (including biochemical and botanicals) and microbial pesticides. (10) The Reg. EC 1107/2009 defines the two-step regulatory process of a biopesticide that includes: (*i*) evaluation and approval of the active substances (a.s.) at the EU level, followed by the (*ii*) evaluation of PPPs in zonal level (3 administrative EU zones: northern, central, southern) and authorization by the concerned Member States. The regulatory process steps of evaluation are made based on the following requirements: *a*) sufficiently effective to control a target disease/pest on the specific crop(s); *b*) impact on human and animal health; *c*) Fate and behavior in the environment (*e.g.*, persistence, bioaccumulation, potential for long-range environmental transport, ecotoxicology, residue definition, impact on non-target species, impact on biodiversity and the ecosystem).

As regards Asian Countries, particularly China, the Institute for the Control of Agrochemicals of the Agriculture Ministry is responsible for the registration and regulation of biopesticides. They are broadly categorized into six categories: botanical, microbial, biochemical, biological, genetically modified organisms (GMOs), and agro-antibiotics based on Chinese data requirements for pesticide registration (Wang et al., 2022). In India, the regulatory centers are represented by the Central Integrated Pest Management Centre (CIPMC) at Faridabad and the National Centre for IPM (NCPM) under the Indian Agricultural Research Council; on the other hand, the Directorate of Biological Control and the marketing of biopesticides to farmers is under the responsibility of the Ministry of Agriculture and Farmers Welfare and the Department of Biotechnology (DBT) (Chakraborty et al., 2023). Meanwhile, in Africa, several nations use a variety of guidelines to create systems for the registration and control of biopesticide regulation aimed at the management of diseases. A regional inventory of the regulatory environments was conducted by six African countries as West African regions (Kenya, Uganda, Ethiopia, Tanzania, Nigeria) and Ghana, as part of the Commercial Products (COMPRO II) project, which is overseen by the International Institute of Tropical Agriculture (IITA) (Arora et al., 2016).

Moreover, different authorities (*i.e.*, the EU Commission, European Food Safety Authority, Competent Evaluation and Authorization Authorities of the 27 EU Member States) are involved in the biopesticides evaluation and authorization processes. This results in a laborious and more prolonged process (approval procedure of a.s. ~ 2–4 years, followed by those of new PPPs ~ 1–2 years) compared with the United States, South America, and Asia (Karamaouna et al., 2023). Moreover, when the previous evaluation system of the EU was based on regulation 91/414/EEC and then followed by directive 1107/2009, just 26% of registered Plant Protection Products (PPP) and active substances were annulled in agriculture (Nawaz et al., 2023). Arora et al. (2016) highlighted another complex issue surrounding the regulation of biopesticides having multiple modes of action (*i.e.*, biofertilizer/phytostimulators and biocontrol agents), as in the case of fluorescent Pseudomonads. They can be sold based on their plant growth-promoting properties rather than as plant protection products, escaping scrutiny from regulators regarding their efficacy and safety because there are no particular regulatory mechanisms to check agronomic efficacy and the risks associated with human, animal or plant health or to the environment before biofertilizer commercialization. Indeed, while the European Union and countries like Brazil, India, and China have made progress in this area, the USA market lacks clear regulation guidelines for biofertilizer production and sale, highlighting the lack of a globally coordinated uniform regulatory policy (Santos et al., 2024).

## 4 Commercial biopesticides

During the last few years, a limited number of new chemical formulations have been marketed by the industrial sector as a consequence of the low perceived market value of conventional bactericides and the uncertainty of acquiring registration for plant disease management (Sharma et al., 2020). Moreover, the cost and time associated with the development process of new chemical bactericides have been a significant barrier to commercialization: it can exceed $250 million and take over a decade to bring the drugs to the field (Ma et al., 2023). In contrast, biopesticide development cost ranges from $5–25 million, with a time to market of 5–7 years (https://croplife.org/wp-content/uploads/2024/02/Time-and-Cost-To-Market-CP-2024.pdf, accessed on 21 September 2024). In this regard, biopesticides require less intensive regulatory scrutiny and leverage existing fermentation and formulation technologies, resulting in lower overall costs that make biopesticides a more attractive option for industrial companies and growers (Samada and Tambunan, 2020). Although biopesticides are eco-friendly, reducing harmful residues and promoting biodiversity, regionally variable regulatory frameworks, efficient formulations and application technologies remain significant challenges to their commercialization and use (Butu et al., 2022).

Regarding the formulation of commercial biopesticides, it poses important challenges to guarantee the development of high-quality preparations with stable shelf life and proven efficacy products that can be implemented in the field for potential marketability. Indeed, factors such as temperature, moisture, UV radiation and certain plant-produced chemicals can negatively impact the viability and efficacy of biopesticides, leading to an increase in the application frequency with an increase in costs (Buttimer et al., 2017). Other fundamental aspects to be taken into consideration for the most efficient formulations are the ecology and biology of biopesticides, the pathosystem, the environment and the application niche, the inoculation techniques (*e.g.*, foliar spray, soil spray, soil drench, soil irrigation, seed coating) and types of irrigation systems (*e.g.*, sprinkler irrigation, drip irrigation) involved in the cropping system (Bejarano and Puopolo, 2020).

Nowadays, conventional biopesticide formulations are usually based on very few variants: powder, granulated or liquid forms, with a wide range of carrier materials, protectants, and optimized delivery systems, which can facilitate their integration into comprehensive disease management programs for vegetable growers (Bonaterra et al., 2022). Commercially accessible biocontrol solutions that control bacterial plant disease are still few and in the beginning phases (Lahlali et al., 2022). However, for these commercial biopesticides, the several technological challenges listed above were surmounted. A list of some commercially available biocontrol products against *Pseudomonas* spp. and *Xanthomonas* spp. bacterial diseases of vegetable crops are documented in **Table 1**, together with trade names, crops, target pests, territories marketed and web links. The majority of commercial EOs are characterized by their direct antibacterial action, such as cell wall destruction, telomerase inhibition, cell membrane damage leading to loss of cytoplasmic content and ergosterol depletion against the bacterial pathogens affecting tomato, pepper, bean, cabbage and cauliflower. For EOs, common application strategies resulting in efficient control of bacterial diseases are based on ground sprays, aerial foliar sprays and to soil or crops through irrigation systems (*i.e.*, chemigation).

**Table 1:** A broad description of some commercial biopesticides applied in the control of *Pseudomonas* spp. and *Xanthomonas* spp. bacterial diseases on tomato and pepper*, Fabaceae* and *Brassicaceae*.

Category	Name/Active Principle	Host	Pathogen	Mode of Action	Territories marketed	Web link
EOs	Guarda^®^ (Thyme oil, Thymol chemotype)	Tomato, Pepper, *Brassicaceae, Fabaceae*	*Xanthomonas* spp.*, Pst*	Antibiosis	USA	Guarda^®^
EOs	Sporan EC2^®^ (Rosemary, clove, peppermint, and thyme oils)	Tomato, Pepper, *Brassicaceae, Fabaceae*	*Xanthomonas* spp.*, Pst*	Antibiosis	USA	Sporan EC2^®^
EOs	GreenFurrow BacStop^®^ (*clove, rosemary, peppermint, cottonseed*, *thyme, garlic, cinnamon*)	Tomato, Pepper	*Xanthomonas* spp.	Antibiosis	USA (Exempt from EPA registration)	GreenFurrow BacStop^®^
*m*BCAs	Serenade ASO^®^ (*Bacillus subtilis* QST 713)	Tomato, Pepper, *Brassicaceae*	*Xanthomonas* spp., Pst	Competition, ISR, Antibiosis	USA, EU	Serenade ASO^®^
*m*BCAs	Rhapsody*^®^* (*Bacillus subtilis* QST 713)	Tomato, Pepper, *Brassicaceae*, Bean, Pea	*Xanthomonas* spp.*, Pst, Pseudomonas* spp.	Competition, ISR	USA	Rhapsody^®^
*m*BCAs	Cease*^®^* (*Bacillus subtilis* QST 713)	Tomato, Pepper, *Brassicaceae*	*Xanthomonas* spp.*, Pst*	Competition, Antibiosis	USA	Cease^®^
*m*BCAs	Amylo-X^®^ (*Bacillus amyloliquefaciens* subsp. *plantarum* D747)	Tomato, Pepper, *Brassicaceae*	*Xanthomonas* spp.*, Pst*	Competition, ISR, Antibiosis	USA, EU	Amylo-X^®^
*m*BCAs	Double Nickel 55^®^ (*Bacillus amyloliquefaciens* D747)	Tomato, Pepper, *Brassicaceae*	*Xanthomonas* spp.*, Pst*	Competition	USA; Canada	Double Nickel 55^®^
*m*BCAs	Taegro^®^ 2 (*Bacillus subtilis* var. *amyloliquefaciens* FZB24)	Tomato, Pepper	*Xanthomonas* spp.	Competition, ISR, Antibiosis	USA; Canada	Taegro^®^2
*m*BCAs	Stargus^®^ (*Bacillus amyloliquefaciens* F727)	Tomato, Pepper	*Xanthomonas* spp.	Competition, Antibiosis	USA; Canada	Stargus^®^
*m*BCAs	Serifel^®^ (*Bacillus amyloliquefaciens* MBI 600)	Tomato, Pepper, *Brassicaceae*,	*Xanthomonas* spp.*, Pst*	Multiple mode of action	USA, EU	Serifel^®^
*m*BCAs	AmyloShield™ *(Bacillus amyloliquefaciens* PTA-4838)	Tomato, Pepper, *Brassicaceae, Fabaceae*	*Xanthomonas* spp.*, Pst*	Antibiosis (cyclic lipopeptides)	USA; Canada	AmyloShield™
*m*BCAs	Companion*^®^* (*Bacillus subtilis* GB03)	Tomato, Pepper, *Brassicaceae*, Bean, Pea	*Xanthomonas* spp.*, Pst, P. syringae*	Competition, ISR	USA	Companion^®^
*m*BCAs	Baciforte^®^ (*Bacillus subtilis* C55)	Tomato	*Pst*	Competition, Antibiosis	USA	Baciforte^®^
*m*BCAs	AVIV^®^ (*Bacillus subtilis* IAB/BS03	Tomato, Pepper, *Brassicaceae*	*Xanthomonas* spp.*, Pst*	Competition, ISR, Antibiosis	USA	AVIV^®^
*m*BCAs	LifeGard^®^ (*Bacillus mycoides* isolate J)	Tomato, Pepper, *Brassicaceae, Fabaceae*	*Xanthomonas* spp.*, Pst*	ISR	USA	LifeGard^®^
*m*BCAs	*NACILLUS ^®^* *(Bacillus subtilis* isolate *Antumávida, Bacillus subtilis* isolate *Vilcún, Bacillus licheniformis* isolate *Mallerauco, Brevibacillus brevis* isolate *aguellines*, *Brevibacillus brevis* isolate *Maguellines I)*	Tomato, Cabbage	*Pst, Xv*	Competition, Antibiosis	Chile	Nacillus^®^
*m*BCAs	BlightBan A506*^®^* (*Pseudomonas fluorescens* A506)	Tomato	*Pst*	Competition	USA	BlightBan A506^®^
*m*BCAs	Howler EVO^®^ (*Pseudomonas chlororaphis* AFS009)	Tomato, Pepper, *Brassicaceae*	*Xanthomonas* spp.*, Pst*	Competition, ISR, Antibiosis	USA	Howler EVO^®^
*m*BCAs	Actinovate AG^®^ (*Streptomyces lydicus* WYEC 108)	Tomato, Pepper	*Xanthomonas* spp.*, Pst*	Competition, Chitinase and siderophore production	USA; Canada	Actinovate AG^®^
*phage*BCAs	AgriPhage^®^	Tomato, Pepper	*Xv, Pst*	Lysis of the host bacterial cell	USA	Agriphage^®^

The biopesticides are divided in category (*i.e.*, EOs, *m*BCAs or *phage*BCAs), name or active principle, host plant, pathogen, mode of action (*i.e.*, antibiosis, competition and/or ISR), countries marketed and web link of the bioproduct.

Among commercial *m*BCAs for the control of bacterial diseases, twelve bacteria are registered in the EU as active ingredients: *Bacillus amyloliquefaciens* strains QST 713 (formerly *B. subtilis*), AH2, MBI 600, FZB24 and IT 45, *Bacillus amyloliquefaciens* subsp. *plantarum* strain D747, *Bacillus subtilis* strain IAB/BS03, *Pseudomonas* spp. strain DSMZ 13134, *Pseudomonas chlororaphis* strain MA 342, *Streptomyces* K61 and *Streptomyces lydicus* strain WYEC 108 (https://food.ec.europa.eu/plants/pesticides/eu-pesticides-database_en, accessed on 10 October 2024). Meanwhile, eight *m*BCAs are marketed exclusively in USA, Chile, Brazil or Canada: *Pseudomonas fluorescens* strain A506, *Bacillus subtilis* strains C55, GB03, N5, *Brevibacillus parabrevis* strain N4, *Bacillus cereus* strains N6, N7, *Bacillus amyloliquefaciens* strains F727 and PTA-4838, *Bacillus mycoides* strain J and finally the *Pseudomonas chlororaphis* strain AFS009 (https://www.epa.gov/ingredients-used-pesticide-products/biopesticide-active-ingredients, accessed on 11 October 2024) (**Table 1**). These biopesticides are available in several formulations (*e.g.*, liquid, powder, wettable powder, water dispensable granule) and characterized by different modes of actions, such as: (*i*) competition, antibiosis and ISR, provided by Serenade ASO^®^, Amylo-X^®^, Taegro^®^2, Serifel^®^, AVIV^®^ and Howler EVO^®^; (*ii*) competition and ISR, provided by Rhapsody*^®^* and Companion*^®^*; (*iii*) competition and antibiosis, provided by Cease*^®^*, Stargus^®^, Baciforte^®^, Nacillus^®^; (*iv*) competition, provided by Actinovate AG^®^, Double Nickel 55^®^ and BlightBan A506*^®^*; (*v*) ISR, provided by LifeGard^®^ and finally through (*vi*) antibiosis by AmyloShield™.

Different application methods (*i.e.*, seed treatments, soil drench, in-furrow, ground spray, aerial spray, chemigation) during the entire cropping season of tomato, pepper, bean, cabbage and cauliflower are possible. Conversely, the commercial availability of *phage*BCAs is very limited. Among the *Pseudomonas* spp. and *Xanthomonas* spp. considered in this review, only AgriPhage^®^ (OmniLytics, Inc., Sant Lake City, UT, USA) has specific applications for controlling both bacterial spot and speck of tomato and pepper. Its liquid formulation relies on ground and aerial spray applications as preventive treatments when conditions are conducive to heavy disease pressure or when the first disease symptoms are visible. As argued above, the variety of possible formulations has also increased, thanks to the increased technologies in such fields; this made biopesticides more functional to the grower needs and for large-scale employment, provided by supposed lower costs of production. The liquid or dry formulations have been improved in conservation characteristics, and they may have longer life-shelf than that in the past, at different temperature conditions; that partly compensates the high costs of the biopesticides, which in some cases may be triple compared to some conventional copper compounds (Tazzari, 2019).

## 5 Recent publications on novel biopesticide candidates

Systematic literature research from 2019-2024 years survey) was carried out to summarize the most recent progress and the current research trends on identifying and testing BCAs based on EOs, *m*BCAs and *phage*BCAs. The recent studies listed in **Table 2** reported biopesticide candidates for sustainable control of diseases caused by *Pseudomonas* spp. and *Xanthomonas* spp. on vegetable crops, together with their mode of action, experimental assay levels (*i.e.*, *in vitro*, growth chamber, greenhouse or field conditions) and references.

**Table 2:** Recent (2019-2024 years survey) BCAs tested for *Pseudomonas syringae pv. tomato, Pseudomonas savastanoi pv. phaseolicola* and *Xanthomonas* spp. control on tomato and pepper, bean, cabbage and cauliflower.

Category	Name/Active Principle	Host	Pathogen	Mode of Action	Assay level	Reference
EOs	*Eugenol*	Tomato	*Xep*	Antibiosis	Field	(Jibrin et al., 2022)
EOs	*Carvacrol*	Tomato	*Xep*	Antibiosis	Field	(Qiao et al., 2020)
EOs	Lemongrass, oleum and thyme	Tomato	*Xv*	Antibiosis	Greenhouse	(Khalil Bagy and Abo-Elyousr, 2019)
EOs	*Tetraclinis articulata*	Tomato	*Pst*	Antibiosis	Greenhouse	(Es-sahm et al., 2024)
EOs	*Origanum compactum, Citrus aurantium* var. *amara* (hydrolate)	Tomato	*Xv*	ISR	Growth chamber	(Proto et al., 2022)
EOs	*Satureja montana*	Tomato	*Xee*	Antibiosis, ISR	*in vitro*	(Oliveira-Pinto et al., 2022)
EOs	Caraway oil (*Carum Carvi*)	Tomato	*Xv*	Antibiosis	*in vitro*	(Kim et al., 2023)
EOs	*Origanum dubium*	nd	*Xv, Pst, Xaph*	Antibiosis	*in vitro*	(Basim et al., 2019)
EOs	*Elionurus latiflorus Cymbopogon flexuosus*	nd	*Pst, Xaph*	Antibiosis	*in vitro*	(Martinazzo et al., 2022)
EOs	*Clove*	Bean	*Xaph*	Antibiosis	Greenhouse	(Imran et al., 2023)
EOs	*Satureja cuneifolia, Satureja* sp*icigera, Satureja thymbra, Satureja hortensis, Satureja cilicica*	Bean	*Xaph, Xcf*	Antibiosis	Greenhouse	(Dönmez et al., 2022).
EOs	*Origanum heracleoticum, Origanum majorana*	Bean	*Psph*	Antibiosis	*in vitro*	(Della Pepa et al., 2019)
EOs	*Oregan*	Bean	*Psph, Xcf*	Antibiosis	*in vitro*	(Elshafie et al., 2020)
EOs	*Origanum vulgare*	nd	*Psph, Xc*	Antibiosis	*in vitro*	(Gruľová et al., 2020)
EOs	*Laurus nobilis*	nd	*Psph*	Antibiosis	*in vitro*	(Mamoucha et al., 2023)
EOs	*Thyme, Clove (carvacrol, eugenol, linalool, p-cymene and thymol)*	Cabbage	*Xcc*	Antibiosis	Growth chamber	(Hakalová et al., 2022)
EOs	*Croton grewioides Baill*	nd	*Xcc*	Antibiosis	*in vitro*	(Rodrigues et al., 2023)
EOs	*Thymol-loaded chitosan nanoparticles (TCNPs)*	nd	*Xcc*	Antibiosis	*in vitro*	(Sreelatha et al., 2022)
EOs	*Lippia gracilis*	nd	*Xcc*	Antibiosis	*in vitro*	(Da Silva et al., 2019)
EOs	*Cordia curassavica Jacq. (baleeira herb)*	nd	*Xcc*	Antibiosis	*in vitro*	(Da Silva et al., 2020)
EOs	*Moringa oleifera*	nd	*Xcc*	Antibiosis	*in vitro*	(Fontana et al., 2021)
*m*BCAs	*Bacillus mycoides J (Bmj)*	Tomato	*Xep*	ISR	Field	(Strayer-Scherer et al., 2024)
*m*BCAs	*Pseudomonas umsongensis O26, P. vranovensis A30, P. resinovorans A5, P. resinovorans A28, P. resinovorans A33, P. resinovorans A47, P. brassicacearum N6, P. rassicacearum N32, P. putida T15, P. stutzeri N42, P. putida C21, P. aeruginosa B30, P. alcaligenes B5, P. alcaligenes B16*	Tomato	*Pst*	ISR	Field	(Elsharkawy et al., 2023)
*m*BCAs	*Bacillus amyloliquefaciens* var. *plantarum* D747	Tomato	*Xv*	Competition, ISR, Antibiosis	Greenhouse	(Biondi et al., 2019)
*m*BCAs	*Bacillus cereus F-BC26, Bacillus cereus F-BC08, Bacillus thuringiensis F-BT24*	Pepper	*Xee*	Competition, Antibiosis	Greenhouse	(Hernández-Huerta et al., 2023)
*m*BCAs	*Bacillus velezensis* GF267	Tomato	*Xep*	Competition, ISR, Antibiosis	Greenhouse	(De Paula Kuyat Mates et al., 2019)
*m*BCAs	*Bacillus velezensis* 71, *Paenibacillus peoriae* To99	Tomato	*Xep, Xhg*	Competition, Antibiosis	Greenhouse	(Olishevska et al., 2023)
*m*BCAs	*Streptomyces* spp. AN090126 (culture filtrates)	Pepper	*Xee*	Antibacterial, Antimicrobial VOCs	Greenhouse	(Le et al., 2022)
*m*BCAs	*Priestia megaterium T3*, *Bacillus cereus T4*	Tomato	*Xee*	ISR, Antibiosis	Greenhouse	(Gupta et al., 2023)
*m*BCAs	*Streptomyces* spp. SA51, *Pseudomonas* spp. PT65	Tomato	*Xv*	Competition, Antibiosis	Greenhouse	(Vurukonda et al., 2022)
*m*BCAs	*Herbaspirillum seropedicae HRC54*	Tomato	*Xee*	ISR	Greenhouse	(Da Silva et al., 2021)
*m*BCAs	*Trichoderma viride, Trichoderma harzianum, Trichoderma album, Bacillus subtilis, Pseudomonas fluorescens, Serratia marcescens* (culture filtrates)	Tomato	*Xee, Xv, Pst*	Competition; Antibiosis	Greenhouse	(Akila et al., 2024)
*m*BCAs	*A. leptinellae E138* (culture filtrates)	Tomato	*Pst*	Competition, Antibiosis, Quorum Quenching activities	Greenhouse	(García-Latorre et al., 2024)
*m*BCAs	*Bacillus thuringiensis SE, Bacillus toyonensis EI, Bacillus thuringiensis RA*	Tomato	*Pst*	ISR	Greenhouse	(Es-sahm et al., 2024)
*m*BCAs	*Bacillus velezensis IP22*	Pepper	*Xee*	Competition, Antibiosis	Growth chamber	(Pajčin et al., 2020)
*m*BCAs	*Bacillus* spp.	Pepper	*Xv*	Competition, Antibiosis	Growth chamber	(Aziz et al., 2024)
*m*BCAs	*Pseudomonas simiae POE78A, Bacillus velezensis PSE31B, Leclercia* spp. *S52, Bacillus velezensis PFE11*	Tomato	*Xep*	Competition, Antibiosis	Growth chamber	(Nicotra et al., 2024)
*m*BCAs	*Bacillus amyloliquefaciens CHB 310, Trichoderma asperellum CHF 78*	Tomato	*Xep*	Competition, Antibiosis	Growth chamber	(Chien and Huang, 2020)
*m*BCAs	*Pseudomonas segetis P6*	Tomato	*Pst*	Quorum Quenching activities	Growth chamber	(Rodríguez et al., 2020)
*m*BCAs	*Bacillus amyloliquefaciens MBI600*	Tomato	*Pst*	Competition, Antibiosis	Growth chamber	(Dimopoulou et al., 2021)
*m*BCAs	*Rhizobium b1*, *Bacillus subtilis b2*	Tomato	*Pst*	Competition, ISR, Antibiosis	Growth chamber	(Shao et al., 2023)
*m*BCAs	*100 bacterial and fungal isolates (see paper for detailed list)*	Tomato	*Pst*	Competition, ISR, Antibiosis	Growth chamber	(Köhl et al., 2020)
*m*BCAs	*Pseudomonas koreensis 5, Bacillus mycoides 68, Bacillus mojavensis 36, Bacillus simplex 47*	Tomato	*Pst*	ISR	Growth chamber	(Yildiz et al., 2023)
*m*BCAs	*Trichoderma harzianum GT 3-2, Fusarium equiseti GF 18-3, F. equiseti GF 19-1, Phoma* spp. *GS 10–1 Phoma* spp. *GS 14-1*	Tomato	*Pst*	ISR	Growth chamber	(Elsharkawy et al., 2021)
*m*BCAs	*Burkholderia contaminans AY001*	Tomato	*Pst*	ISR	Growth chamber	(Heo et al., 2022)
*m*BCAs	*Acremonium sclerotigenum 13237*	Tomato	*Pst*	ISR	Growth chamber	(Llorens et al., 2022)
*m*BCAs	*Pantoea agglomerans ZM2, Pantoea agglomerans ZM3, Pantoea dispersa ZM1*	Tomato	*Pst*	Competition, ISR, Antibiosis	Growth chamber	(Morella et al., 2019)
*m*BCAs	*Bacillus subtilis QST 713*	Bean	*Xaph*	Competition, Antibiosis	Greenhouse	(Belete et al., 2021)
*m*BCAs	*Pseudomonas fluorescens A33, Bacillus. simplex Z51, Bacillus. pumilus Z73*	Bean	*Xaph*	Competition, Antibiosis	Greenhouse	(Rostami et al., 2021)
*m*BCAs	*Bacillus alcalophilus ÖSLP3/7*, *Bacillus atrophaeus ZA142, Bacillus subtilis ZA114, Bacillus subtilis ÖBF20, Bacillus megaterium ZA146, Bacillus megaterium ZA129, Bacillus megaterium ZA136, Bacillus mycoides ÖBF21, Lysinibacillus* sp*haericus ZA91, Pseudomonas fluorescens ZA79*, *Pseudomonas putida ÖBF76*	Bean	*Psph*	Competition, Antibiosis	Greenhouse	(Dönmez and Aliyeva, 2023)
*m*BCAs	*Pseudomonas grimontii P25, Pseudomonas cepatia P7*	Bean	*Xaph*	Competition, Antibiosis	Growth chamber	(Mokrani et al., 2019)
*m*BCAs	*Burkholderia gladioli BNM349*	Bean	*Xcf*	Competition, Antibiosis	Growth chamber	(Alvarez et al., 2024)
*m*BCAs	*Trichoderma harzianum T22, Burkholderia gladioli*	Bean	*Psph, Xcf*	Antibiosis	*in vitro*	(Elshafie et al., 2020)
*m*BCAs	*Bacillus* spp.*, Pseudomonas* spp.*, Rhizobium radiobacter, Arthrobacter* spp.*, Achromobacter* sp*anius, Serratia liquefaciens, Acinetobacter calcoaceticus, Exiguobacterium* spp.*, Microbacterium hydrocarbonoxydans, Ochrobactrum anthropi.*	nd	*Psph*	Antibiosis	*in vitro*	(Duman and Soylu, 2019)
*m*BCAs	*Lactobacillus pentosus J02, Leuconostoc fallax J13*	Cabbage	*Xcc*	Competition, Antibiosis	Greenhouse	(Lin et al., 2020)
*m*BCAs	*Trichogin GA IV-derived peptides (Trichoderma longibrachiatum)*	Cauliflower	*Xcc*	Competition, Antibiosis	Greenhouse	(Caracciolo et al., 2023)
*m*BCAs	*Pseudomonas fluorescens CFLB-27 Bacillus velezensis CFLB-24, Bacillus amyloliquefaciens CFLB-31, Stenotrophomonas rhizophila CFLB-26*	Cauliflower	*Xcc*	Competition, Antibiosis	Greenhouse	(Geat et al., 2024)
*m*BCAs	*Trichoderma* spp.*, Pleosporales* spp.*, Fusarium* spp.*, Curvularia* spp., *Setophoma/Edenia* spp.*, Acrocalymma* spp.	(Kale Cabbage)	*Xcc*	ISR	Greenhouse	(Poveda et al., 2020)
*m*BCAs	*Serendipita indica DSM 11827*	Cabbage	*Xcc*	Competition, ISR	Growth chamber	(Saleem et al., 2023)
*m*BCAs	*Paenibacillus polymixa N179*	Turnip Cabbage	*Xcc*	ISR	Growth chamber	(Fallahzadeh-Mamaghani et al., 2021)
*m*BCAs	*Burkholderia anthina HN-8*	Cabbage	*Xcc*	Quorum Quenching activities	*in vitro*	(Ye et al., 2020)
*m*BCAs	*Acinetobacter lactucae QL-1*	Cabbage	*Xcc*	Quorum Quenching activities	*in vitro*	(Ye et al., 2019a)
*m*BCAs	*Cupriavidus* spp. *HN-2*	Cabbage	*Xcc*	Quorum Quenching activities	*in vitro*	(Ye et al., 2019b)
*m*BCAs	*Bacillus velezensis FZB42*	nd	*Xcc*	Antibiosis	*in vitro*	(Mácha et al., 2021)
*m*BCAs	*Bacillus velezensis M 5*	nd	*Xcc*	Antibiosis	*in vitro*	(Grahovac et al., 2021)
*m*BCAs	*Bdellovibrio bacteriovorus SOIR-1*	nd	*Xcc*	Antibiosis	*in vitro*	(Odooli et al., 2021)
*phage*BCAs	*Bacteriophages KF1*	Pepper	*Xee*	Lysis of the host bacterial cell	Field	(Šević et al., 2019)
*phage*BCAs	*Bacteriophages PL4, S4 and GF2*	Tomato	*Xep*	Lysis of the host bacterial cell	Greenhouse	(De Sousa et al., 2023)
*phage*BCAs	*Bacteriophage Medea1*	Tomato	*Pst*	Lysis of the host bacterial cell, Induced resistance	Greenhouse	(Skliros et al., 2023)
*phage*BCAs	*Bacteriophages ΦXp06-02-1* (*in combination with NAC-ZnS*)	Pepper	*Xep*	Lysis of the host bacterial cell	Greenhouse	(Choudhary et al., 2023)
*phage*BCAs	*Bacteriophage BsXeu269p/3*	Pepper	*Xee*	Lysis of the host bacterial cell	Growth chamber	(Shopova et al., 2023)
*phage*BCAs	*Bacteriophage P1*, *Bacteriophage P2*	Tomato	*Pst*	Lysis of the host bacterial cell	Growth chamber	(Hernandez et al., 2020)
*phage*BCAs	*Bacteriophage D6*	Tomato	*Pst*	Lysis of the host bacterial cell	Growth chamber	(Wu et al., 2024)
*phage*BCAs	*Bacteriophage* Eir4, *Bacteriophage* Eisa	Pepper	*Pst*	Lysis of the host bacterial cell	*in vitro*	(Korniienko et al., 2022)
*phage*BCAs	*Bacteriophage XaF13*	Pepper	*Xee*	Lysis of the host bacterial cell	*in vitro*	(Solís-Sánchez et al., 2020)
*phage*BCAs	*Bacteriophage* XaC1, *Bacteriophage* XbC2	Tomato	*Xv*	Lysis of the host bacterial cell	*in vitro*	(Villicaña et al., 2024)
*phage*BCAs	*Ps virus-1, Ps virus-2, Ps virus-3, Ps virus-4, Ps virus-5, Ps virus-6*	Bean	*Psph*	Lysis of the host bacterial cell	Field	(Faiesal et al., 2023)
*phage*BCAs	*Bacteriophage B1, Bacteriophage B21, Bacteriophage BV72, Bacteriophage T12, Bacteriophage T21*	Bean	*Psph*	Lysis of the host bacterial cell	*in vitro*	(Martino et al., 2021)
*phage*BCAs	*Bacteriophage DB1*	Cabbage	*Xcc*	Lysis of the host bacterial cell	Greenhouse	(Orynbayev et al., 2020)
*phage*BCAs	*Bacteriophage Murka*	nd	*Xcc*	Lysis of the host bacterial cell	*in vitro*	(Evseev et al., 2024)
*phage*BCAs	*Bacteriophage Xccφ1* (*in combination with 6PP and HA*)	nd	*Xcc*	Biofilm formation	*in vitro*	(Papaianni et al., 2020)

The biopesticides are divided in category (EOs, *m*BCAs or *phag*eBCAs), name or active principle, host plant, pathogen, mode of action (*i.e.*, antibiosis, competition and/or ISR), experimentation scale (*i.e.*, *in vitro*, growth chamber, greenhouse or field) and the reference of the publication.

EOs are basically obtained through hydro- or steam distillation of plant tissues and by cold pressing of *Citrus* spp. fruit peel; a co-product of the distillation is the hydrolate, which stands for aromatic water containing approx. 0.1% EOs mixture (Di Vito et al., 2019). To date, the hydrolates are basically studied for their activities against human bacterial pathogens, since Proto et al. (2022) evaluated their direct *in vitro* activity against Xv. Nowadays, the majority of studies on EOs concern the evaluation of the antimicrobial activity *in vitro* against plant bacterial pathogens. In several works it was highlighted the ability of EOs extracted from plant species such as *Thymus* spp., *Origanum* spp., *Eucalyptus* spp., or *Mentha* spp. among others, in inhibiting the *in vitro* growth through diffusion or dilution methods against Pst, Xv, Xee on tomato, Xcf and Psph on bean, and Xcc on cabbage, or in affecting the capability to produce biofilms by observations with scanning electron microscopy (SEM) of Xv (Proto et al., 2022), Pst and Psph (Della Pepa et al., 2019; Jamshidi et al., 2023). Other studies took into account, again *in vitro*, the antimicrobial activity of carvacrol and thymol, or other essential oils extracted by *Moringa oleifera* against Xcc and by observing the impact of the EOs on Xcc vitality, motility, and biofilm’s formation on cabbage seeds (Hakalová et al., 2022). Concerning the activity against Xaph on beans, few studies have evaluated the efficacy of EOs obtained by *Satureja* spp. and clove in significantly inhibiting the pathogen growth under greenhouse experiments (Dönmez et al., 2022; Imran et al., 2023). Likewise, different EOs have highlighted their antibacterial capability to control pathogen infections *in planta* for Xep, Xv, Pst on tomato (Es-sahm et al., 2024; Jibrin et al., 2022; Khalil Bagy and Abo-Elyousr, 2019; Qiao et al., 2020), and for Xcc on cabbage (Hakalová et al., 2022) and on radish (*Raphanus sativus* L.) (Fontana et al., 2021). Besides the *in vivo* antibacterial activity of EOs described above, Proto et al. (2022) demonstrated the potential of *Origanum compactum* and a hydrolate obtained from *Citrus aurantium* by acting through the plant host and triggering the ISR: root treatments of tomato plants significantly reduced the bacterial leaf spot severity caused by experimental inoculation of Xv with respect to untreated control plants. On beans, Imran et al. (2023) showed the production of eugenol in plants treated with clove oil, which acts against Xaph through both direct pathogen inhibition and enhancing the plant immune system by triggering the expression level of PR proteins.

Bacterial pathogens penetrate the plant through natural openings (*e.g.*, stomata, hydathodes) or through wounds, where organs of the plant are equally susceptible to the plant pathogen survival and the subsequent penetration into the host (Akhavan et al., 2013). Therefore, EOs are studied to prevent the penetration of plant bacterial pathogens and to avoid the following infection of the host (DuPont et al., 2023). Unfortunately, they have shown problems with their stability and persistence on the plant organ’s surfaces, primarily due to their rapid evaporation and their unequal coverage of the treatment. Besides, the increase in the EO’s concentration may provoke phytotoxic effects on the organs (Borges et al., 2018; Proto et al., 2022). This is the reason why EOs need co-formulants able to improve the distribution and reduce the evaporation of the active principle (Proto et al., 2022). In fact, various solid and liquid formulations of EOs have been assayed to enhance their handling, stability, and controlled release. Innovative “green” solid formulations for encapsulation, such as microencapsulation and nanoparticles, offer prolonged and sustained release of active compounds compared to their liquid counterparts (Dunan et al., 2023). In two recent studies, EOs have been employed against Xcc in combination with other antimicrobial agents, such as chitosan nanoparticles, highlighting the potential of these carriers for the delivery of EO biopesticides for effective plant disease management (Sreelatha et al., 2022). Likewise, liquid nanoemulsions have demonstrated a superior ability to enhance the stability, water solubility, and biological activity of EOs, making them more effective than their direct application (López et al., 2021).

In **Table 2**, *Bacillus* spp. and *Pseudomonas* spp. have been the most widely studied microorganisms in controlling diverse bacterial phytopathogens through their multiple modes of action, including resource competition, antibiosis and ISR. Solely two studies have been conducted under field conditions, both of them on tomato, by testing the ISR potential of *Bacillus mycoides* isolate J (the active ingredient of the biofungicide LifeGard^®^ WG; Marin et al., 2019; Strayer-Scherer et al., 2024; Tariq et al., 2020) and *Pseudomonas* spp (Elsharkawy et al., 2023). The biopesticide *Bacillus mycoides* J resulted not effective in controlling Xep by foliar treatments, nor in combination with Kocide^®^ 3000 (copper hydroxide); meanwhile, *Pseudomonas resinovorans* A47, *Pseudomonas brassicacearum* N32, and *Pseudomonas putida* T15 showed a considerable decrease in bacterial speck severity in comparison to Pst control, by triggering the SA immune response pathway and increasing the peroxidase and polyphenol oxidase activities. In a greenhouse study, root treatments with *Bacillus amyloliquefaciens* spp. *plantarum* strain D747 (active principle of Amylo-X^®^ and Double Nickel 55^®^) upregulated the SA signaling pathway in tomato plants. There, the phytopathometric assessment showed a significant reduction of the bacterial spot Xv pathogen severity at levels similar to those of streptomycin sulfate and acibenzolar-S-methyl (ASM) treated plants (Biondi et al., 2019). *Streptomyces* spp. and *Bacillus* spp. were also investigated for their capability to produce volatile organic compounds (VOCs), such as dimethyl sulfide and trimethyl sulfide, and cyclic lipopeptides in the culture filtrates, which were able to significantly inhibit the growth of Xee compared to streptomycin sulfate and untreated controls in *in planta* assays, respectively (Le et al., 2022; Pajčin et al., 2020).

Besides, *Trichoderma* spp. were the most tested fungal *m*BCAs, mainly investigated against tomato and cabbage bacterial diseases under growth chamber and greenhouse conditions. These experiments evaluated the production and excretion of fungal secondary metabolites and antimicrobial peptides present in the culture filtrates against Xee, Xv, Pst and Xcc (Akila et al., 2024; Caracciolo et al., 2023). Additionally, different *Trichoderma* spp. have proved their ability to elicit ISR on tomato and cabbage plants and reduce Pst and Xcc severity compared to untreated control plants, respectively (Elsharkawy et al., 2021; Poveda et al., 2020). Other fungal *m*BCAs were promising in controlling bacterial diseases by up-regulating defense pathways dependent on SA and/or JA signaling pathways, such as *Fusarium equiseti* and *Phoma* spp. against Pst, and *Serendipita indica* against Xcc (Elsharkawy et al., 2021; Saleem et al., 2023).

Notably, a promising strategy has been studied for controlling diseases by *quorum quenching* (QQ) mode of action. QQ involves the enzymatic degradation of N-acyl homoserine lactones (AHLs) signal molecules attenuating virulence and reducing the infection of bacterial pathogens (Uroz et al., 2009). Under greenhouse conditions, Rodríguez et al. (2020) showed the QQ potential of *Pseudomonas segetis* strain P6 on tomato plants infected by Pst. In *in vitro* assays, QQ biocontrol mechanisms were reported for the bacterial isolates *Acinetobacter lactucae* QL-1 (Ye et al., 2019a), *Cupriavidus* spp.HN-2 (Ye et al., 2019b), *Burkholderia anthina* HN-8 (Ye et al., 2020) against Xcc and the fungal isolate *Alternaria leptinellae* E138 against Pst (García-Latorre et al., 2024).

Among *phage*BCAs, their efficacy has been reported in different papers, underscoring satisfactory results on the control of bacterial spot and speck, halo blight, and black rot. They described results obtained *in vitro* or in growth chamber experiments on *phage*BCAs isolation, characterization and testing for the lysis ability against Xv (Villicaña et al., 2024), Xee (Shopova et al., 2023; Solís-Sánchez et al., 2020), Pst (Hernandez et al., 2020; Wu et al., 2024), Psph (Martino et al., 2021) and Xcc (Evseev et al., 2024). *In vitro*, the bacteriophage Xccφ1, alone or formulated in combination with 6-pentyl-α-pyrone (6PP) and hydroxyapatite (HA) nanocrystals, proved the capability to interfere with the gene pathways involved in the formation of Xcc biofilm. 6PP, produced by *Trichoderma atroviride*, was tested for its antibiotic and plant metabolome interference properties; instead, HA nanocrystals were assessed for bacteriophage delivery and as an enhancer of its biological activities and stability (Papaianni et al., 2020).

In greenhouse experiments, spray application of *phage*BCAs resulted in disease reduction of pepper bacterial spot (*i.e.*, Xep) at levels higher than those achieved by the use of copper hydroxide (De Sousa et al., 2023). For Pst, Skliros et al. (2023) reported the *Pseudomonas* phage Medea1 biocontrol potential through its (*i*) lytic nature and ability (*ii*) and by upregulating SA and abscisic acid (ABA) defense pathways by foliar spray and root drenching applications, respectively. During a four-year study, *phage*BCAs were assessed in combination with copper hydroxide and acibenzolar-S-methyl (ASM) as a part of integrated disease management practices in pepper fields, improving the efficacy of single *phage*BCAs and providing a consistent bacterial spot control compared to plants sprayed with water only (Šević et al., 2019).

*Phage*BCAs offer promising biocontrol potential in agriculture, as highlighted by the above-described studies. However, the most significant limiting environmental factor is the phage’s vulnerability to UV light, which can drastically reduce their viability. Nonetheless, recent studies highlighted the effectiveness of *phage*BCA formulations with skimmed milk and corn flour, or riboflavin, to control Psph and Xcc compared to plants sprayed with copper or water, respectively, under greenhouse and open field conditions (Faiesal et al., 2023; Orynbayev et al., 2020). All these examples of studies on EOs and *m*BCAs highlight the increased need for biopesticides and, at the same time, the increased variability of available BCAs, which implicitly might prevent treatments based only on one or two strains or naturals. It is clear that the complete protection of the crop from bacterial diseases is challenging; applying integrated control methods, from the seed diagnostic analyses to the chemical compounds treatment to the biopesticides employment and to optimal agronomical measures, will not be possible. The increase in biopesticide employment, in any case, avoids the accumulation of toxic compounds at the soil level, and the increased variety may enrich the soil microflora, being environmentally advantageous. Furthermore, nano-formulations of N-acetyl cysteine zinc sulfide (NAC-ZnS) has shown to significantly improve both UV stability and the antimicrobial efficacy of bacteriophages against the tomato bacterial spot pathogen Xep, making them more effective at sunlight exposure (Choudhary et al., 2023).

## 6 Perspectives and future challenges

The gap between studies on BCAs and their translation into commercial products in the EU, for instance, might be mainly determined by the inadequacy of BCAs, characterized at the laboratory level, to all the requirements of the registration process, such as eco-toxicological risks. Another possible answer might be found in the inefficacy of BCAs applied in the field or in the greenhouse under real-scale conditions, which may be due to the poor attention that the scientific community gives to the research focused on their production, formulation and delivery (Bejarano and Puopolo, 2020). Furthermore, other main limitations comparing the full adoption of biopesticides are the high cost of the commercial products compared to the available conventional agrochemical products, the inability to meet the global market demand and the extreme variability of the methods used for the bio-formulations (Fenibo et al., 2021).

Bio-formulation is, indeed, one of the critical points for the gap between research studies and real field conditions, as discussed above for EOs. In fact, the evaporation of the EO active principle strongly limits the stability and persistence of the treatment that aims to directly inhibit pathogen survival at the epiphytic phase and its penetration into the host. To overcome the persistence limit, some new commercially available formulations of EO mixtures are composed of a recently patented micro-clay (Patent N° EP 3071039-28.09.2016), amended with a low percentage of heavy metal as copper or zinc to enhance the effectiveness of the EOs. Other attempts to employ nano-technology were carried out against Xcc in *in vitro* assays by evaluating the effect of Thymol-Loaded Chitosan Nanoparticles against the pathogen (Sreelatha et al., 2022). Against other bacterial pathogens, such as *Xanthomonas fragariae* on strawberry, these combined formulations have demonstrated their efficacy, either *in vitro* and *in planta* experiments, under controlled and field conditions (Biondi et al., 2022).

To strengthen the efficacy of *m*BCA applications, innovative formulation approaches are being developed to improve microbial stability, shelf life, and field performance, ensuring their viability and effectiveness under field conditions (Saberi Riseh et al., 2022). Research and companies involved in biopesticide development are exploring formulations made by means of emulsions, encapsulations, hydrogels, and nanoproducts, each of which offers distinct advantages for microbial stability and controlled release in agricultural applications. Nanoformulations and microencapsulation technologies, in particular, have been shown to improve the residual action of biopesticide, potentially expanding their practical field use by increasing persistence in agriculture conditions (Damalas and Koutroubas, 2018; Hernandez-Tenorio et al., 2022). Alginate-based microcapsules have demonstrated potential for encapsulating plant biocontrol bacteria due to their biocompatibility, biodegradability, and capacity to support long-term microbial survival. However, more studies are needed to verify their efficacy in disease management (Saberi Riseh et al., 2021). Biopolymer-based formulations, such as those combining polydopamine particles with whey protein isolates, have been shown to significantly improve both UV stability and the antimicrobial efficacy of *phage*BCAs, making them more effective in agricultural environments (Huang and Nitin, 2020). For *m*BCAs, what really lacks are studies on their survival in the host organs over a long period, as up to one month, in order to well define the persistence of the active alive principle.

Besides the formulation challenges, the compatibility among BCAs represents an additional criticism in the framework of integrated and non-integrated pathogen management. In particular, the compatibility between EOs and other BCAs has not yet been studied, neither *in vitro*: the risk, in this case, could be the inhibition of *m*BCA by EOs treatment (*e.g.*, negative effect of EOs toward the bacterial biofilm formation). This important aspect can also directly affect the colonization, survival and persistence of the *m*BCA in the cropping systems (Sreelatha et al., 2022). Considering the compatibility among multiple *m*BCA strains or species *in vitro*, different rapid methods are available to assess their possible coexistence and synergy (Vanneste et al., 1992). Under greenhouse or field conditions, on the contrary, the time of the experiments is significantly longer, and several times, there were no positive correlations between the *in vitro* and *in planta* results. Thus, the first screening rounds using *in vitro* tests on a large number of isolates followed by *in planta* testing of a selected group of candidates may not exploit the entire potential of antagonists. For instance, microbial antagonists combining various modes of action may be excluded by *in vitro* screening with a bias on a specific mode of action (Köhl et al., 2020).

## 7 Conclusions

The diseases caused by the genera *Pseudomonas* and *Xanthomonas* on tomato, pepper, bean, cabbage and cauliflower include several bacterial species affecting the crops yearly and causing significant economic losses in various countries. Chemical control of these plant pathogenic bacteria still results in problems and ineffectiveness, not providing a solution to plant infection or disease eradication. Moreover, the adverse effects on human health and the environment stressed the importance of ecological alternatives for managing plant pathogenic *Pseudomonas syringae* pv*. tomato, Pseudomonas savastanoi* pv*. phaseolicola and Xanthomonas* spp. Nowadays, the cost and time for developing new chemical bactericides have been a significant barrier to commercialization compared to developing biopesticides. Nevertheless, commercially accessible BCAs for managing bacterial diseases are few in the beginning phases. Among commercial *m*BCAs, only a few bacteria are registered in the EU as active ingredients, and others are marketed exclusively in the American continent. For EOs, three products are available for the market in the USA, significantly limiting their availability worldwide. So far, for the antibacterial products based on *phage*BCAs, the commercially available product is one in the USA, against bacterial speck and spot of tomato and pepper. Studies on EOs, *m*BCAs, and *phage*BCAs in the last five years have shown promising but variable results, with only a few trials conducted in field conditions. The lack of a uniform regulatory model for pesticide development, registration, and use is a significant barrier to the widespread use of biopesticides. This non-uniformity underscores the urgent need for policy changes that can simplify the registration process and promote the use of biopesticides. Such changes could facilitate the translation of academic research into practical agricultural solutions, potentially revolutionizing pest management in agriculture.
